# *HIM*-herbal ingredients *in-vivo* metabolism database

**DOI:** 10.1186/1758-2946-5-28

**Published:** 2013-05-31

**Authors:** Hong Kang, Kailin Tang, Qi Liu, Yi Sun, Qi Huang, Ruixin Zhu, Jun Gao, Duanfeng Zhang, Chenggang Huang, Zhiwei Cao

**Affiliations:** 1 School of Life Sciences and Technology, Tongji University, Shanghai, 200092, China; 2 Shanghai Center for Bioinformation and Technology, 1278 Keyuan Road, Shanghai, 201203, China; 3 College of Information Engineering, Shanghai Maritime University, Shanghai, 201306, China; 4 Shanghai Institute of Materia Medica, Shanghai Institute for Biological Science, Chinese Academy of Sciences, Shanghai, 201203, China

**Keywords:** TCM, *In vivo*, Metabolism, Metabolite, Biotransformation, Structure search

## Abstract

**Background:**

Herbal medicine has long been viewed as a valuable asset for potential new drug discovery and herbal ingredients’ metabolites, especially the *in vivo* metabolites were often found to gain better pharmacological, pharmacokinetic and even better safety profiles compared to their parent compounds. However, these herbal metabolite information is still scattered and waiting to be collected.

**Description:**

*HIM* database manually collected so far the most comprehensive available *in-vivo* metabolism information for herbal active ingredients, as well as their corresponding bioactivity, organs and/or tissues distribution, toxicity, ADME and the clinical research profile. Currently *HIM* contains 361 ingredients and 1104 corresponding *in-vivo* metabolites from 673 reputable herbs. Tools of structural similarity, substructure search and Lipinski’s Rule of Five are also provided. Various links were made to PubChem, PubMed, TCM-ID (Traditional Chinese Medicine Information database) and HIT (Herbal ingredients’ targets databases).

**Conclusions:**

A curated database *HIM* is set up for the *in vivo* metabolites information of the active ingredients for Chinese herbs, together with their corresponding bioactivity, toxicity and ADME profile. *HIM* is freely accessible to academic researchers at http://www.bioinformatics.org.cn/.

## Background

As one of the naturally originated medical systems, Chinese herbal medicine (CHM) has developed for several thousand years and accumulated plenty of clinical experiences and pharmacological information to form its own integrated theory system [1]. Being a multi-component and multi-target therapy methodology, the studies on its molecular mechanism have made a great progress in recent years although much more still remains unclear [2-4]. In order to get a deeper insight into the mechanism of CHM, various modern scientific technologies have been applied to separate and purify the active ingredients from herbs and elucidate their pharmacodynamic characteristics [5]. Over the past few years, many active compounds have been separated and their pharmacological effects were tested [6-8]. More interestingly, during many researches [9-11], active metabolites were sometimes found to gain better pharmacological, pharmacokinetic and safety profiles compared to their respective parent compounds. For example, morphine is a widely used analgesic which was extracted from Papaver somniferum L and its major therapeutic benefit is mediated by morphine-6-glucuronide, an active metabolite of morphine [12,13]. Another example, Ginsenoside-Rb1, a major active ingredient of Panax ginseng, is found to have the antiallergic activity through its main metabolite named compound K instead of itself [14]. Similarly, glycyrrhizic acid, an alicyclic compound which was extracted from Glycyrrhiz glabra L, has no effect of anti-lipid peroxidation in rat hepatocyte, while its metabolite, glycyrrhetinic acid, has the inhibitory effect on lipid peroxidatioin in dose-dependent manner [15]. Many other similar instances can be found which implies an important message that metabolites of herbal ingredients could be highly valuable for new drug discovery.

Currently, abundant metabolism information of herbal active ingredients has been produced with the progress of TCM modernization. However, although there exist several databases such as MDL Metabolite Database [16] and Accelrys Metabolism Database [17] for synthetic compounds whose pharmacokinetic and metabolism data have been carefully stored, there is still lack of specific database to collect and store the corresponding information for herbal active ingredients. It is noted that synthetic compounds metabolic databases have made great contributions to new drug discovery [18]. Constructing a database collected the CHM ingredients metabolism information could also have substantial positive impact on TCM development.

In our previous work, in order to collect available resources of protein targets for FDA-approved drugs and the promising precursors, we developed HIT [19] (http://lifecenter.sgst.cn/hit/). Served as a serial work after HIT, HIM is a database which aims to provide the systematical and accurate data storage, data access as well as data analysis (i.e., structural similarity search and substructure analysis) for the herbal active ingredients *in vivo* metabolism information. In this work, the *in vivo* metabolism data of those active ingredients extracted from herbs were collected from literature, unpublished in-house experimental data and the Chinese herbal medical monographs. The information from all these heterogeneous data sources was further processed and integrated into a well-designed database. The information of each ingredient was divided into three categories: identification label, metabolic scheme and bioactivity information. Additionally, properties like the number of hydrogen bond (H-bond) donors and acceptors, molecular weight or the octanol-water partition coefficient logP, which allow the evaluation of the Lipinski’s Rule of Five, can be found within the database. The 2D structures of all the compounds are available and the structure similarity search function and substructure search function are also provided. In summary, up to now, there are 361 active ingredients from 673 Chinese herbs and 1104 corresponding metabolites stored in HIM. All the data were freely accessible at http://www.bioinformatics.org.cn/ for academic researches.

## Construction and content

### Data source

The data in HIM were compiled from both primary and secondary sources.

First, Chinese herbal ingredients *in vivo* metabolism data were extracted from PubMed [20] literature by searching with key words: metabolism, metabolite, biotransformation, metabolic, CHM, Chinese herbal medicine, *in vivo*. Then a preliminary screening was carried out by browsing all the abstracts manually. After that we checked the full text for all the qualified articles and extracted the information according the database criterion. At last, the data is confirmed when they passed the quality control process which consists of rechecking and revising. In summary, about one-third of all entries come from literatures.

Second, metabolism data were extracted from the book entitled “Absorption, Distribution, Metabolism, Excretion, Toxicity and Activity of The Chemical Constituents in Traditional Chinese Medicines” [21]. This book is a well-known TCM monograph which is concerning ADME/T (Absorption, Distribution, Metabolism, Excretion and Toxicity) of CHM active ingredients in China. Approximately half of the entries in the database are derived from this book, which made such valuable information available online for the first time.

Third, some unpublished *in vivo* experimental data about CHM ingredients metabolism are also gathered in HIM, which accounts for the remaining minority of the entries.

### Content and details

The database HIM comprises three data fields for each active ingredient: identification label, metabolic scheme and bioactivity information.

#### Identification label

In this field, following information is provided for each record:

Common, Alias and Systematic Names. Both the Chinese pinyin and the common English name are provided. The aliases of each active ingredient that are obtained from the database SciFinder [22] are also listed. The systematic name presents precise details of a chemical structure which is generated based on the IUPAC names of natural product skeletal types.

CAS Registry Number. CAS number provides a reliable link between different systems of nomenclature as well as an access to future information on every ingredient. In addition, the compounds are also annotated with the CID numbers provided by PubChem [23] with a hyperlink to it.

Botanical Species. Latin binomials of the herbs and the corresponding region of the plant in which the ingredient located are listed.

#### Metabolic scheme

In this field, detailed information about *in vivo* metabolism data of each CHM active ingredient is available.

Molecular Weight and Molecular Formula. The MW and MF of all compounds are given by using the functions provided by Marvin Bean [24], a JAVA package supplied by ChemAxon [25].

Structure. For each CHM active ingredient and its metabolites, a 2D structure which is stored in MDL mol format in HIM is shown in JPEG format on the web page.

Metabolite and Metabolic Scheme. All the metabolites of each active ingredient and the full view of *in vivo* metabolic process are provided in HIM.

#### Bioactivity information

In this field, extensive information about the pharmacokinetic (ADME) properties, bioactivity and toxicity of each active ingredient are listed.

#### Bioactivity

Some general concepts like anti-cancer, anti-inflammation, anti-bacteria, etc. rather than the diseases-related protein targets are used to describe the bioactivity of each active ingredient.

#### Toxicity

As being generally recognized, LD50 (median lethal dose) value is used to represent the toxicity with some concrete descriptions for each ingredient.

**Figure 1:**
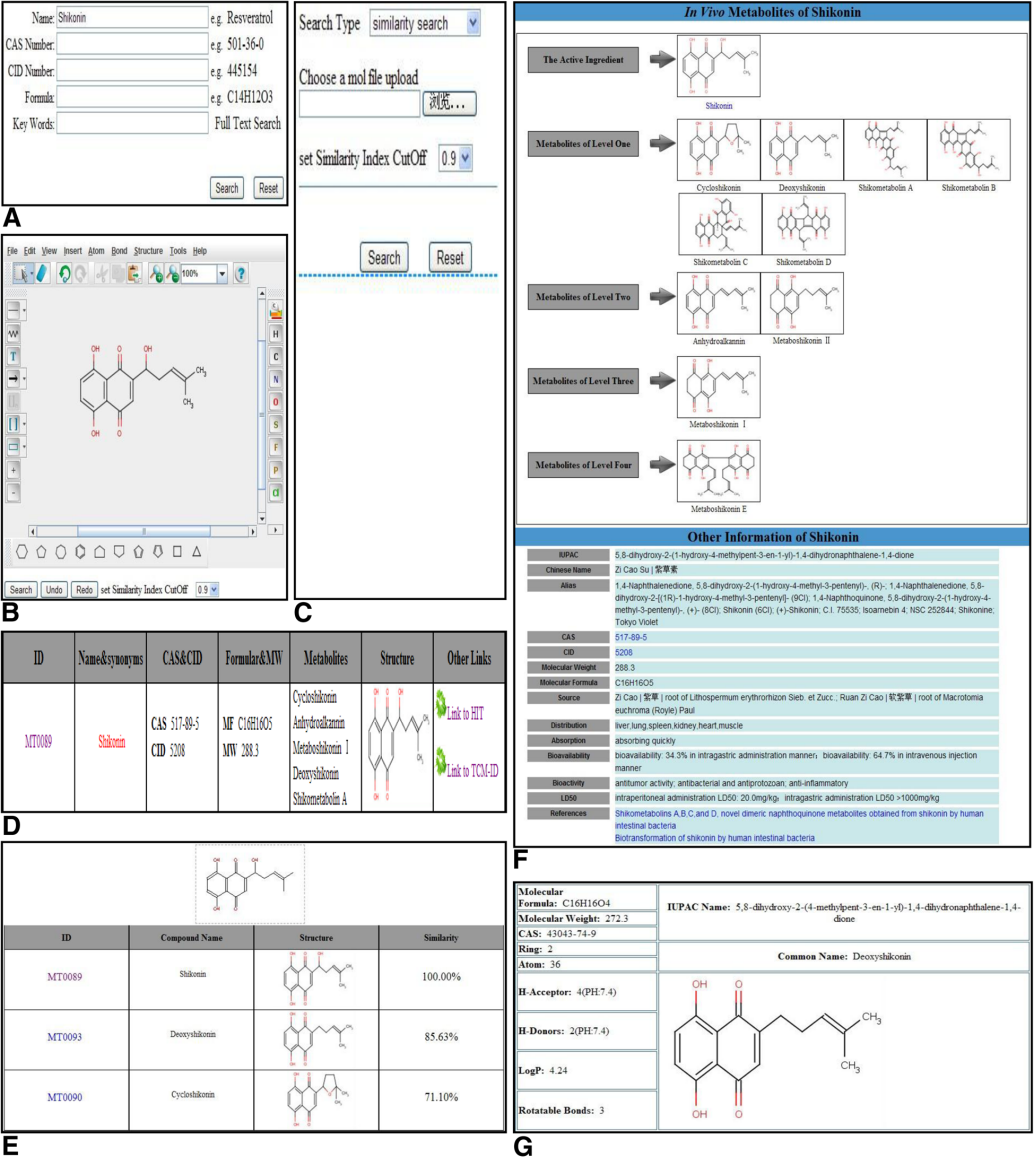
**Primary pages in HIM.** (**A**) Keywords Text Search (**B**) Draw a molecular structure for structure search (**C**) Upload a MOL/SDF file for structure search (**D**) Result page of ‘Text Search’ with ‘Name: Shikonin’. (**E**) Result page of ‘Structure Similarity Search’ with the structure of the compound: Shikonin. (**F**) Detail Information of the compound: Shikonin (**G**) Lipinski’s Rule of Five properties for one metabolite of Shikonin.

Other Information. Besides the bioactivity and toxicity, some other information such as absorption, distribution, clinical research and the main references are also available (see Figure 1).

### Additional functions

#### Text search

The “Text Search” function in the homepage of the website provides five distinctive items to search the whole database: Compound name, CAS Number, CID Number, Molecular Formula and Keywords.

#### Structure similarity search

The “Structure Similarity Search” can be done by uploading a compound structure in MOL/SDF format or via drawing the structure as you want with an embedded molecule editor applet, Marvin Sketch [24]. The structure similarity search is performed by using the so-called structural fingerprint, a binary string with a length of 1024 bits which has encoded the structure characteristics of a given compound. Note that the fingerprint is generated by the Chemistry Development Kit (CDK) [26]. Then the Tanimoto coefficient is calculated by the background program. A molecule with a Tanimoto coefficient ≥ 0.85 to an active compound is often assumed to own similar biological activity [27].

#### Substructure search

Chemical substructure-based *in silico* techniques have been wildly used as an effective and popular approach to reduce the cost in identifying molecules suitable for pharmaceutical development in early stage of drug discovery [28,29]. In our database *HIM*, substructure search is also available by *JChem*[30].

#### Website and server

*HIM* is available online at: http://www.bioinformatics.org.cn/. It is designed as a relational database and implemented in MySQL Server 5.0 with the Apache Tomcat 6.0 as the web server. For chemical calculation and structure drawing, *CDK package* and *Marvin Sketch applet* are embedded. The website is built in JSP, HTML and CSS.

## Utility

HIM (http://www.bioinformatics.org.cn/), which is proposed in this work, is served as a serial work after HIT and concerns about the herbal active ingredients with explicit *in vivo* metabolism data, since the active metabolites of CHM were sometimes found to gain better pharmacological, pharmacokinetic and safety profiles compared to their respective parent compounds. With the help of HIM, researches could find out the mechanism of pharmacological action of CHM more comprehensively, which is expected to have a substantial positive impact on CHM development.

## Discussion

**Figure 2:**
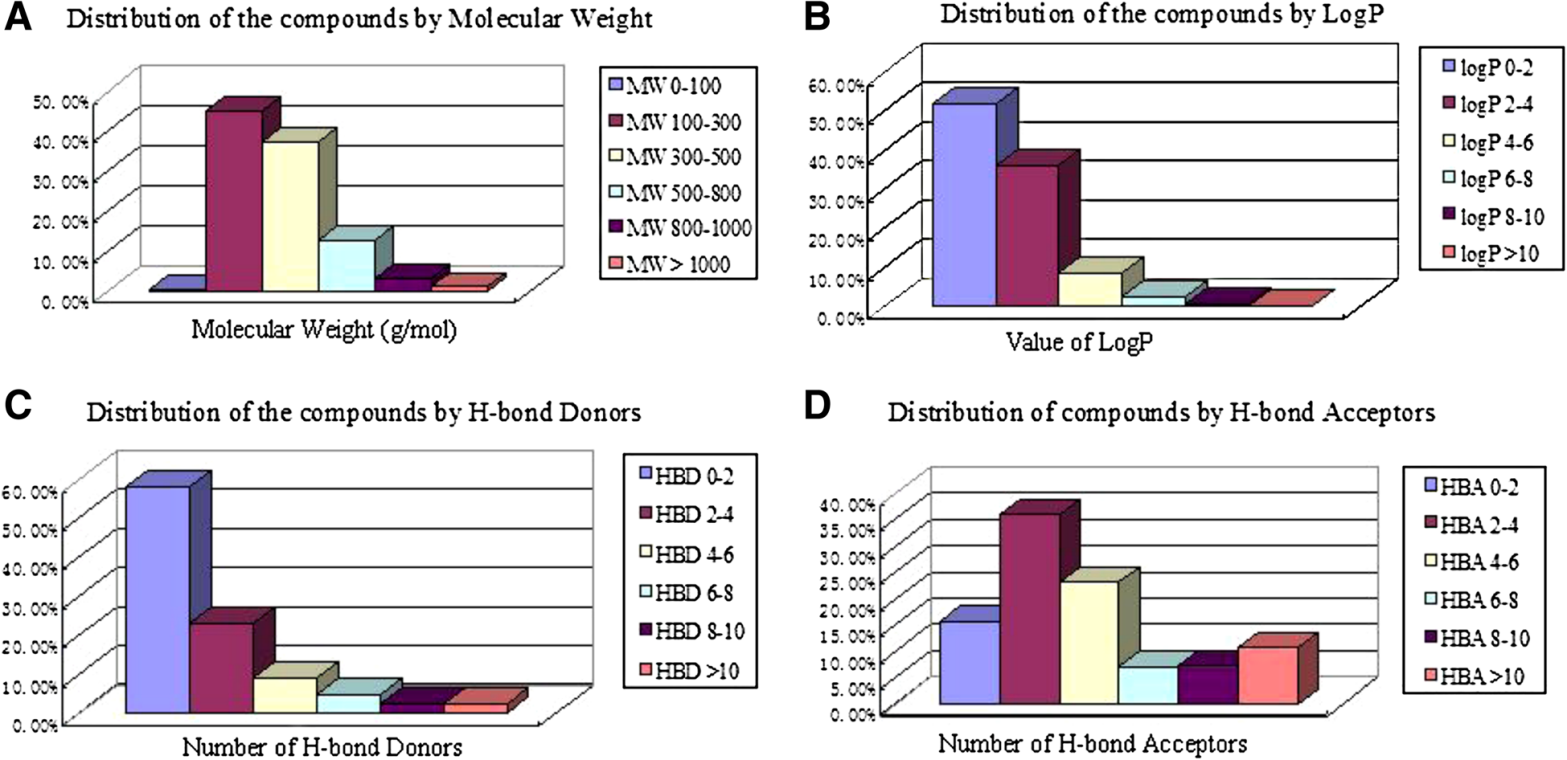
**Lipinski’s rule of five properties statistics.** (**A**) Molecular Weight (**B**) Octanol-water Partition Coefficient logP (**C**) H-bond Donors (**D**) H-bond Acceptors.

Although having been used for thousands of years and own outstanding reputation, the mechanism of CHM is still largely unknown. One reason contributing to this is that it is unclear for the process of ADME/T *in vivo* such as *in vivo* metabolism of CHM. *HIM* is constructed as the first database to store almost all the CHM active ingredients *in vivo* metabolism data dated to January of 2012, as well as their corresponding bioactivity, toxicity, and ADME profile. The properties of Lipinski’s rule of five for each compound are also given to the whole database. As one of the common rules, Lipinski’s rule of five is widely used in drug screening and design. Blake’s [31] study has shown that for the five stages from pre-clinical to approved, less and less compounds break the Lipinski’s rules. It is indicated that compounds which are against the Lipinski’s rule need to be modified too much and they are little probability to be a drug. In *HIM* about 90% of the compounds (herbal ingredients and their metabolites) meet the Lipinski’s rule (Figure 2). We hope that *HIM* can be served as a valuable database to make the progress of CHM modernization and provide great assistance in the new drug discovery and developments.

## Conclusion

*HIM* can be used to get the metabolites of the active ingredient which the researchers are interested in. The structure similarity search and substructure search can be applied to get compounds which potentially similar bioactivity to the query compound and can provide other chemical and biologic information of the query molecule. Moreover, the database is useful for the study of pharmacognosy. Although some active metabolic intermediates are unstable and hard to get, fermentation technology such as microbial transformation could be used to obtain the active compounds which are the *in vivo* metabolites of CHM. Crude herbal medicines could be fermented by certain microbial strains and get certain products [32,33]. *HIM* could provide valuable information for the researchers who are interested in TCM, drug design, pharmacognosy, drug metabolism, etc.

## Competing interests

All the authors declare that they have no competing interests.

## Authors’ contributions

HK compiled the database and developed the web server. DF Z, YS and QH performed database curation and drafted the manuscript. QL, KL T, and RX Z participated in the design of the database and assisted in the manuscript. JG and GQ Z provided guidance and design decisions during the development of the web site and its use cases. CG H, ZW C conceived the study, participated in the design of the database and edited the manuscript. All authors read and approved the final manuscript.
